# Economic evaluation of pegylated interferon plus ribavirin for treatment of chronic hepatitis C in Thailand: genotype 1 and 6

**DOI:** 10.1186/s12876-016-0506-4

**Published:** 2016-08-05

**Authors:** Nattiya Kapol, Surasit Lochid-amnuay, Yot Teerawattananon

**Affiliations:** 1 Department of Community Pharmacy, Faculty of Pharmacy, Silpakorn University, Nakhon Pathom, Thailand 73000; 2 Health Intervention and Technology Assessment Program (HITAP), Ministry of Public Health, Nonthaburi, Thailand

**Keywords:** Chronic hepatitis C, Economic evaluation, Pegylated interferon, Ribavirin

## Abstract

**Background:**

Pegylated interferon alpha 2a, alpha 2b and ribavirin have been included to the National List of Essential Medicines (NLEM) for treatment of only chronic hepatitis C genotypes 2 and 3 in Thailand. This reimbursement policy has not covered for other genotypes of hepatitis C virus infection (HCV) especially for genotypes 1 and 6 that account for 30-50 % of all HCV infection in Thailand. Therefore, this research determined whether pegylated interferon alpha 2a or alpha 2b plus ribavirin is more cost-effective than a palliative care for treatment of HCV genotype 1 and 6 in Thailand.

**Methods:**

A cost-utility analysis using a model-based economic evaluation was conducted based on a societal perspective. A Markov model was developed to estimate costs and quality-adjusted life years (QALYs) comparing between the combination of pegylated interferon alpha 2a or alpha 2b and ribavirin with a usual palliative care for genotype 1 and 6 HCV patients. Health-state transition probabilities, virological responses, and utility values were obtained from published literatures. Direct medical and direct non-medical costs were included and retrieved from published articles and Thai Standard Cost List for Health Technology Assessment. The incremental cost-effectiveness ratio (ICER) was presented as costs in Thai baht per QALY gained.

**Results:**

HCV treatment with pegylated interferon alpha 2a or alpha 2b plus ribavirin was dominant or cost-saving in Thailand compared to a palliative care. The ICER value was negative with lower in total costs (peg 2a- 747,718vs. peg 2b- 819,921 vs. palliative care- 1,169,121 Thai baht) and more in QALYs (peg 2a- 13.44 vs. peg 2b- 13.14 vs. palliative care- 11.63 years) both in HCV genotypes 1 and 6.

**Conclusion:**

As cost-saving results, the Subcommittee for Development of the NLEM decided to include both pegylated interferon alpha 2a and alpha 2b into the NLEM for treatment of HCV genotype 1 and 6 recently. Economic evaluation for these current drugs can be further applied to other novel medications for HCV treatment.

## Background

Chronic hepatitis C virus infection (CHC) is a global important health burden [1]. Untreated infected patients may develop chronic liver problems, including hepatitis, cirrhosis and hepatocellular carcinoma (HCC), and progress to premature death finally [2]. World Health Organization (WHO) reported 150–170 million patients infected with hepatitis C virus and caused 350,000 deaths a year [3]. A global prevalence rate of hepatitis C virus (HCV) infection is 2.5 % [4]. In Thailand, a prevalence of CHC is approximately 2.8 % [5]. Additionally, CHC-related treatment influences an economic burden worldwide [6, 7]. HCV can be transmitted through infected blood include blood transfusions, contaminated needles, body piercing, and hemodialysis. Most of CHC patients are asymptomatic or no specific symptoms then the diseases are silently progressed. A blood screening test by determining anti-HCV antibodies and serum HCV RNA level is recommended for a high risk people [8].

HCV has been classified into six major genotypes, which are distributed differently worldwide. HCV genotype 1, 2, and 3 are broadly distributed in North America, Northern and Western Europe, South America, Asia and Australia. Genotypes 4 and 5 are common in Africa and Middle East, whereas genotype 6 is mainly found in Southeast Asia [9]. The treatment regimen of HCV infection depends on virus genotypes. The American Association for the Study of Liver Diseases (AASLD) [10], the Asian Pacific Association for the Study of the Liver (APASL) [8], the European Association for the Study of the Liver (EASL) [11], and the Thai Association for the Study of the Liver (THASL) [12] recommend pegylated interferon alpha 2a or alpha 2b plus ribavirin as a standard treatment for all genotypes of HCV infection. Treatment of CHC aims to improve quality of life and prevent deaths from cirrhosis and carcinoma. Primary achievement of treatment is undetectable HCV RNA (<50 IU/ml) 24 weeks after the end of treatment or sustained virological response (SVR) [8, 10–12].

Although a combination of pegylated interferon and ribavirin is clinically effective for CHC treatment, some patients cannot afford to pay for drug costs. A palliative care is used for those patients. In 2011, cost-effectiveness of pegylated interferon alpha 2a, alpha 2b and ribavirin have been included to the National List of Essential Medicines (NLEM) in Thailand for treatment of only CHC genotypes 2 and 3 in Thailand since they demonstrates the cost saving comparing to the palliative care [13]. This policy decision, however, has not covered for other genotypes of HCV. Among all genotypes, genotypes 2 and 3 are mainly found in Thailand (40 %). Most of the remaining is genotype 1 and genotype 6, accounting for 20-30 % and 10-20 % of all HCV infection, respectively.

Compared to HCV genotypes 2 and 3, pegylated interferon alpha 2a or alpha 2b plus ribavirin produced a greater SVR than in HCV genotype 6 but less than in HCV genotype 1 [14]. However, most economic evaluation studies of CHC treatment were conducted in Europe [15–17], US [18] and South America [19, 20]. No study of the combination of pegylated interferon alpha 2a or alpha 2b and ribavirin for HCV genotypes 1 and 6 in Southeast Asia including Thailand has been investigated. Therefore, this study aimed to determine and compare costs and health outcomes of pegylated interferon alpha 2a or alpha 2b plus ribavirin and a palliative care for treatment of genotype 1 and 6 HCV infection in Thailand.

## Methods

### Study design

**Fig. 1:**
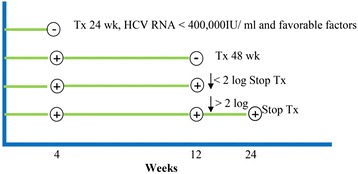
The study treatment guidelines

This study was a model-based economic evaluation. A Markov model was developed from a societal perspective to estimate costs and health outcomes of CHC patients treated with a combination of pegylated interferon alpha 2a or alpha 2b and ribavirin versus a usual palliative care. The study population was a hypothetical cohort of 1000 CHC genotypes 1 or 6 patients. Although, the Thai treatment guidelines (THASL) [12] recommends pegylated interferon and ribavirin combination to an 18-year-old HCV patient, this model cohort assume the patients received treatment at 45 years which is likely to be an average age of current patients in Thailand and other countries [16, 17]. At the beginning of the study, according to THASL guidelines [12], CHC patients genotypes 1 or 6 were 45 years old, a positive RNA test and METAVIR score ≥ 2. The treatment regimens were designed based on THASL guidelines [12] as follows: 1) pegylated interferon alpha-2a 180 μg once a week plus ribavirin 1000 mg/day for 48 weeks, or 2) pegylated interferon alpha-2b 1.5 μg/kg weekly plus ribavirin 800 mg/day for 48 weeks, or 3) a palliative care. SVR rate was assessed 24 weeks after treatment discontinuation, as the achievement. If HCV RNA was undetectable (less than 50 IU/ml) at week 4, defined as a rapid virological response (RVR), the treatment stopped at 24 weeks. If HCV RNA was undetectable at week 12, defined as an early virological response (EVR), the treatment stopped at 48 weeks. If HCV RNA was reduced by less than 2 log at week 12 compared with the baseline level, defined as a null response (NR), the treatment was stopped at week 12. For the remaining cases, we assumed the treatment results as a partial nonresponse (PR), which the treatment was stopped at week 24. Figure 1 presents the study treatment guidelines. In the base-case analysis, costs and health outcomes were discounted at 3 % annually [21].

### Model structure

**Fig. 2:**
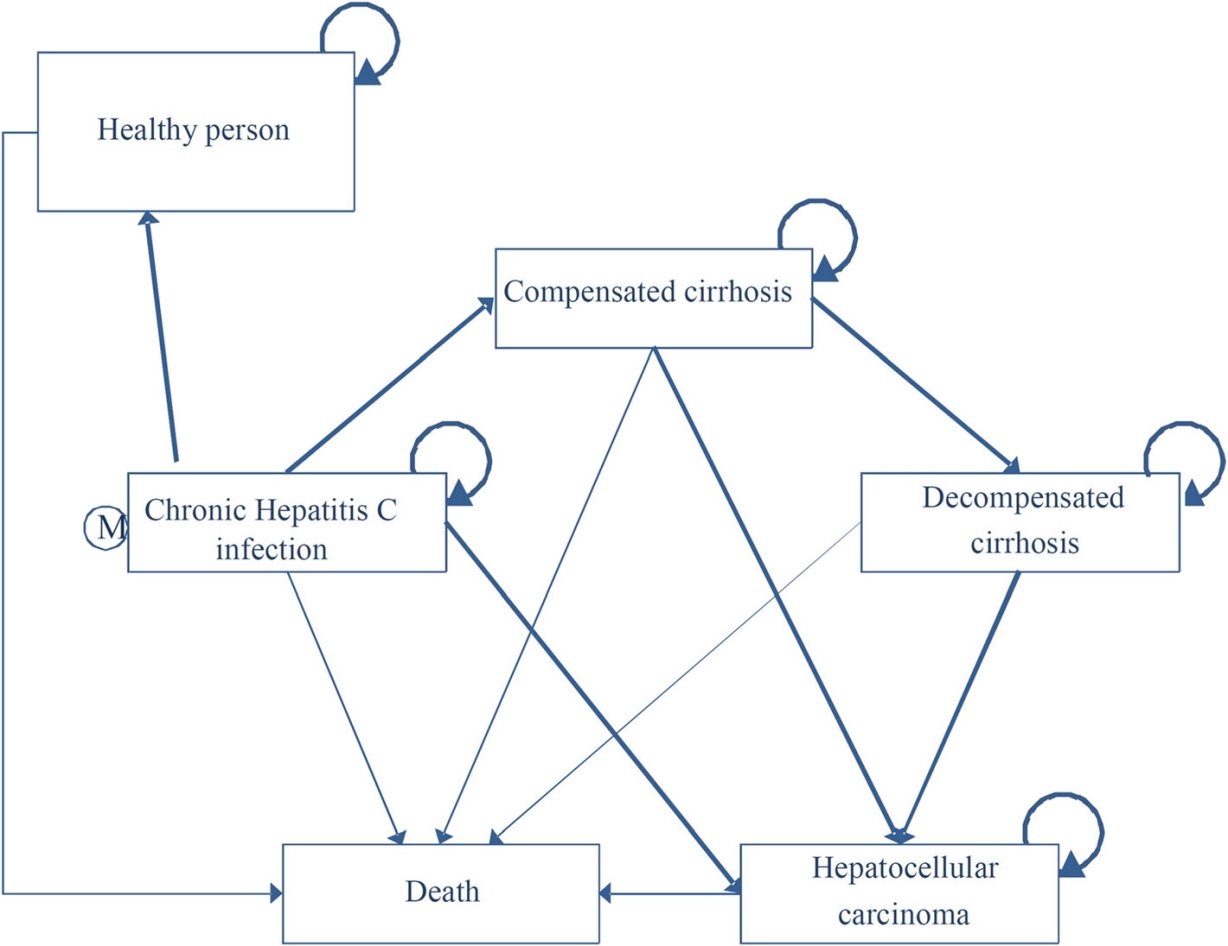
The schematic diagram of the markov model

Figure 2 illustrates the schematic diagram of the Markov model composing of six health states, including CHC, compensated cirrhosis, decompensated cirrhosis, HCC, healthy person, and death. The patients were moved through health states based on transition probabilities. The model started at CHC patients who meet the inclusion criteria for a treatment. If the CHC patients achieved a treatment, they could proceed to the healthy person state, if not, they could continue to the compensated cirrhosis or HCC or death states. The patients in the compensated cirrhosis, decompensated cirrhosis, and HCC could not reverse to CHC or healthy person states. This model assumptions were: 1) the CHC patients weigh sixty kilograms, 2) the patients failure to the treatment are not re-treated, 3) the patients respond to the treatment were not re-infected from HCV, and 4) the patients are 100 % compliant with the treatment. This model was simulated throughout the patients’ lifetime with a 1-year cycle length.

### Model parameters

The parameters used in the model were health state transition probabilities, efficacy data, utility data, and cost data. Health state transition probabilities and utility data were based on the study by Werayingyong, P. and Teerawattananon, Y [13], which conducted a systematic review from published literatures and a meta-analysis of CHC patients’ utility according to their health states (Table 1). Efficacy data of treatment with a combination of pegylated interferon alpha 2a or alpha 2b and ribavirin versus a usual palliative care for CHC genotype 1 were retrieved from a meta-analysis study [22] which included randomized controlled trial studies. According to the expert meeting, the inclusion criteria of RCT studies recruited in the study were publishing after 2008, and reporting SVR and RVR. Finally, the SVR and RVR from three RCT studies, and NR from two RCT studies were pooled to calculated for SVR, RVR and NR. Additionally, Thai general population death rates at each age were used in the analysis [23]. For a 45 year old patient, probability of death from other causes was 0.0044 and varied throughout the patient’s lifetime. All input parameters used in the model presented in Table 1.

**Table 1:** Input parameters used in the model

Input parameters	Mean	Standard error	Distribution	Ref.
Transition probability parameters				
Genotype 1				
chronic HCV to compensated cirrhosis year 1-10	0.0057	0.0057	Beta	[24]
chronic HCV to compensated cirrhosis year 11-20	0.0143	0.0141	Beta	[24]
chronic HCV to compensated cirrhosis year 21-30	0.0207	0.0203	Beta	[24]
chronic HCV to HCC year 1-10	0.0007	0.0007	Beta	[24]
chronic HCV to HCC year 11-20	0.0032	0.0032	Beta	[24]
chronic HCV to HCC year 21-30	0.0063	0.0062	Beta	[24]
chronic HCV to death	0.0070	0.0070	Beta	[25]
compensated cirrhosis to decompensated cirrhosis year1-3	0.0417	0.0400	Beta	[26]
compensated cirrhosis to decompensated cirrhosis year 4-5	0.0945	0.0855	Beta	[26]
compensated cirrhosis to decompensated cirrhosis year 6-10	0.0662	0.0618	Beta	[26]
compensated cirrhosis to HCC year 1-3	0.0135	0.0133	Beta	[26]
compensated cirrhosis to HCC year 4-5	0.0356	0.0344	Beta	[26]
compensated cirrhosis to HCC year 6-10	0.0297	0.0288	Beta	[26]
compensated cirrhosis to death year 1-3	0.0135	0.0133	Beta	[26]
compensated cirrhosis to death year 4-5	0.0461	0.0439	Beta	[26]
compensated cirrhosis to death year 6-10	0.0461	0.0439	Beta	[26]
decompensated cirrhosis to HCC	0.0681	0.0635	Beta	[27]
decompensated cirrhosis to death year 1	0.2600	0.1924	Beta	[24]
decompensated cirrhosis to death year 2	0.3900	0.2379	Beta	[24]
decompensated cirrhosis to death year 3-5	0.2394	0.1821	Beta	[24]
HCC to death year 1	0.8482	0.0011	Beta	[28]
HCC to death year 2	0.9201	0.0009	Beta	[28]
Genotype 6				
chronic HCV to compensated cirrhosis year 1-10	0.0057	0.0057	Beta	[24]
chronic HCV to compensated cirrhosis year 11-20	0.0143	0.0141	Beta	[24]
chronic HCV to compensated cirrhosis year 21-30	0.0207	0.0203	Beta	[24]
chronic HCV to HCC year 1-10	0.0007	0.0007	Beta	[24]
chronic HCV to HCC year 11-20	0.0032	0.0032	Beta	[24]
chronic HCV to HCC year 21-30	0.0063	0.0062	Beta	[24]
chronic HCV to death	0.0070	0.0070	Beta	[25]
compensated cirrhosis to decompensated cirrhosis year 1-3	0.0417	0.0400	Beta	[26]
compensated cirrhosis to decompensated cirrhosis year 4-5	0.0945	0.0855	Beta	[26]
compensated cirrhosis to decompensated cirrhosis year 6-10	0.0662	0.0618	Beta	[26]
compensated cirrhosis to HCC year 1-3	0.0135	0.0133	Beta	[26]
compensated cirrhosis to HCC year 4-5	0.0356	0.0344	Beta	[26]
compensated cirrhosis to HCC year 6-10	0.0297	0.0288	Beta	[26]
compensated cirrhosis to death year 1-3	0.0135	0.0133	Beta	[26]
compensated cirrhosis to death year 4-5	0.0461	0.0439	Beta	[26]
compensated cirrhosis to death year 6-10	0.0461	0.0439	Beta	[26]
decompensated cirrhosis to HCC	0.0681	0.0635	Beta	[27]
decompensated cirrhosis to death year 1	0.2600	0.1924	Beta	[24]
decompensated cirrhosis to death year 2	0.3900	0.2379	Beta	[24]
decompensated cirrhosis to death year 3-5	0.2394	0.1821	Beta	[24]
HCC to death year 1	0.8482	0.0011	Beta	[28]
HCC to death year 2	0.9201	0.0009	Beta	[28]
Virological responses				
Genotype 1				
Pegylated interferon alpha 2b and Ribavirin				
Probability of SVR	0.2720	0.0057	Beta	[29–31]
Probability of RVR	0.1302	0.0033	Beta	[29–31]
Probability to change from RVR to SVR	0.8620	0.1190	Beta	[29]
Probability of NR	0.2449	0.0055	Beta	[29, 31]
Pegylated interferon alpha 2a and Ribavirin				
RR of SVR (peg 2a vs peg 2b)	1.1950	0.0492	Log normal	calculated
RR of RVR (peg 2a vs peg 2b)	1.1300	0.0353	Log normal	calculated
RR of NR (peg 2a vs peg 2b)	0.6960	0.1134	Log normal	calculated
Genotype 6				
RR of SVR (gen 6 vs gen 1)	1.2200		Log normal	[32]
RR of RVR (gen 6 vs gen 1)	1.6800		Log normal	[32]
Health utility				
Chronic hepatitis C infection	0.7284	0.0011	Beta	[13]
Compensated cirrhosis	0.7023	0.0020	Beta	[13]
Decompensated cirrhosis	0.5774	0.0020	Beta	[13]
Hepatocellular carcinoma	0.5778	0.0023	Beta	[13]
SVR (Healthy)	0.7955	0.0018	Beta	[13]

The costs of CHC treatment composed of direct medical costs and direct non-medical costs. To avoid double counting for utility outcomes, indirect costs were excluded. Direct medical costs included drugs (pegylated interferon and ribavirin), laboratory tests (investigation and monitoring) and complication treatment. The prices of pegylated interferon and ribavirin were obtained from the prices that the drug companies presented to the NLEM committee. The types and the number of laboratory tests used in the model were estimated by the expert panel. The tests for investigation included complete blood count (CBC), liver function test, prothrombin time, genotype, viral load, HIV, creatinin, thyroid-stimulating hormone, antinuclear antibodies, abdominal ultrasound, pregnancy test, alanine aminotransferase (ALT), and aspartate aminotransferase (AST). For monitoring, CBC, creatinin, ALT and viral load were included.

Direct non-medical costs included transportation and food expenditure of patients. The number of OPD visits, IPD admissions and length of stay were retrieved from the existing study conducted in Thailand [33]. The unit costs of transportation and food expenditure were derived from Thai Standard Cost List for Health Technology Assessment [34]. All costs were converted in 2013 values by using consumer price index (CPI) and discounted at a rate of 3 %. The average exchange rate of Thai baht (THB) to 1 $US was 35 Baht (Table 2).

**Table 2:** Costs of CHC treatment

Cost parameters	Mean	Standard error	Distribution	Ref.
Medication, laboratory and diagnostic tests costs				
Pegylated interferon alfa-2a + Ribavirin (per week)	3,150	630	Gamma	[35]
Pegylated interferon alfa-2b + Ribavirin (per week)	3,150	630	Gamma	[35]
Investigation and monitoring	16,277	3,255	Gamma	[34]
Direct medical cost for complication treatment				
Costs of chronic HCV infection (per year)	65,640	19,723	Gamma	[33]
Costs of compensated cirrhosis (per year)	73,532	18,605	Gamma	[33]
Costs of decompensated cirrhosis (per year)	138,141	18,996	Gamma	[33]
Costs of hepatocellular carcinoma (per year)	168,899	11,601	Gamma	[33]
Direct non-medical cost for complication treatment				
Costs of chronic HCV infection (per year)	4,303	430.3	Gamma	[33, 34]
Costs of compensated cirrhosis (per year)	4,216	421.6	Gamma	[33, 34]
Costs of decompensated cirrhosis (per year)	5,823	582.3	Gamma	[33, 34]
Costs of hepatocellular carcinoma (per year)	9,516	951.6	Gamma	[33, 34]

Model structure and all parameters were approved by the experts during the expert consultation meeting.

### Sensitivity analysis

A one-way sensitivity analysis and a probabilistic sensitivity analysis (PSA) were performed to determine the uncertainty of model parameters. For one-way sensitivity analysis, each parameter was varied at a time across the plausible range and shown graphically as a tornado diagram. In addition, PSA were carried out by varying all parameters randomly within the plausible range. The Monte Carlo Simulation was generated in order to randomly select a value of each parameter for 1000 times and calculated for expected cost and outcome. The results of PSA were presented by a cost-effectiveness plans and acceptability curves.

## Results

The total costs and QALYs gained of all treatments in patients with HCV genotypes 1 and 6 infection were presented in Table 3. Comparing to palliative care treatment, HCV treatment with pegylated interferon alpha 2a or alpha 2b plus ribavirin both in HCV genotypes 1 and 6 provided the negative ICER value that mean higher in outcomes and lower in costs. The results showed a superior of pegylated interferon alpha 2a plus ribavirin over pegylated interferon alpha 2b plus ribavirin in both genotype 1 and 6. In addition, pegylated interferon plus ribavirin provided less cost and higher outcome in HCV genotype 6 than genotype 1.

**Table 3:** Total costs and QALYs gained

Alternative treatment	Genotype 1			Genotype 6		
	Total costs (Baht)	QALYs (Year)	ICER (compare to palliative care)	Total costs (Baht)	QALYs (Year)	ICER (compare to palliative care)
Palliative care	1,169,121	11.63	-	1,150,417	11.67	-
Peg 2a + RBV	747,718	13.44	Dominant	558,868	14.07	Dominant
Peg 2b + RBV	819,921	13.14	Dominant	655,697	13.69	Dominant

**Fig. 3:**
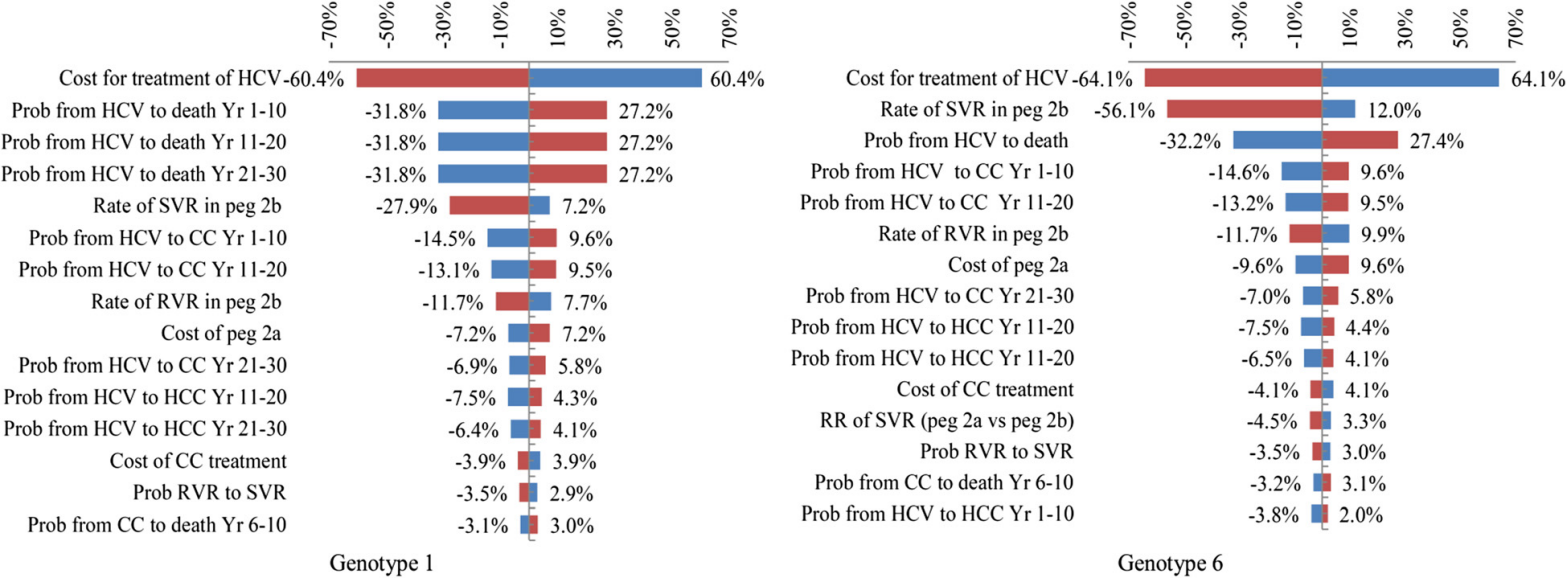
Tornado diagram

The one-way sensitivity analysis of the most cost-saving intervention was presented by a tornado diagram (Fig. 3). Only fifteen parameters most influencing on the model’s results of each HCV genotype was shown from the greatest to the least. For genotype 1, they were as follows: cost of HCV treatment, rate of SVR, transitional probability from HCV state to death, and transitional probability from HCV state to compensated cirrhosis state.

**Fig. 4:**
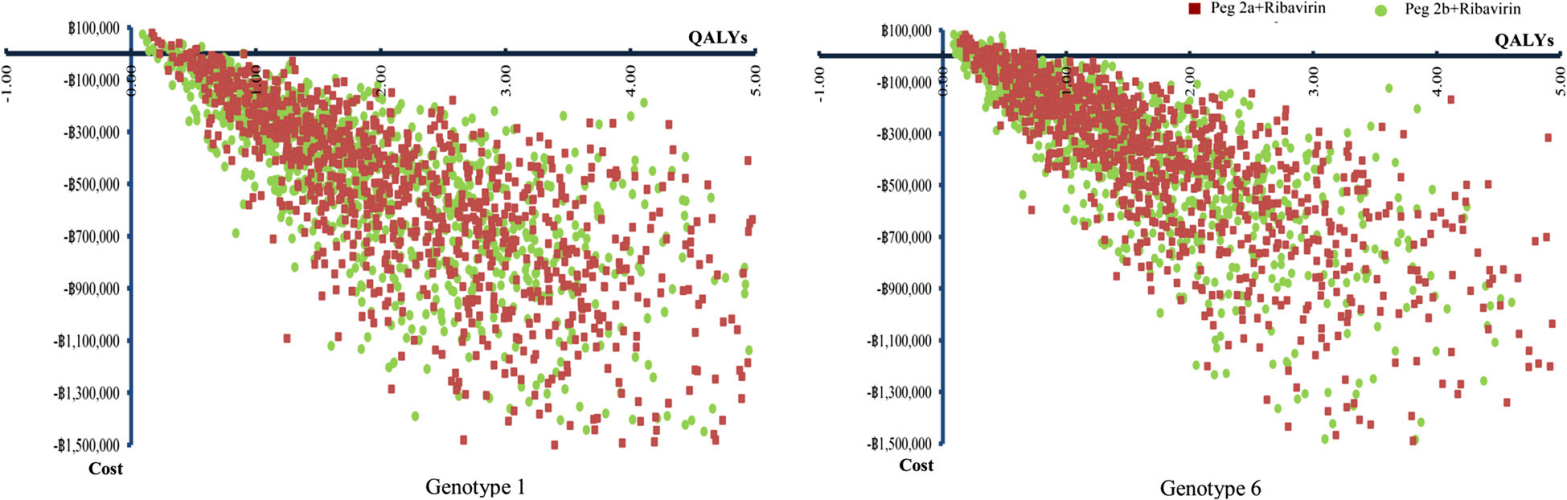
Incremental cost-effectiveness plane

**Fig. 5:**
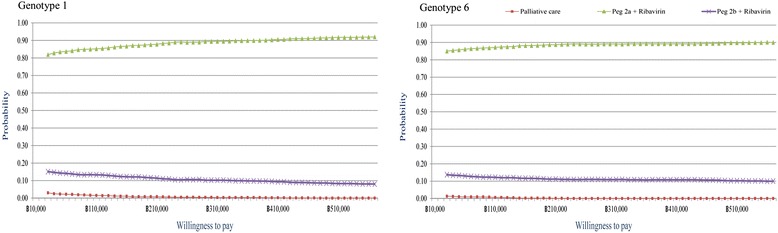
Acceptability curve

The PSA results of both HCV genotypes 1 and 6 treatment were illustrated by cost-effectiveness plane (Fig. 4) and acceptability curves (Fig. 5). The cost-effectiveness acceptability curves showed the superior of pegylated interferon alpha 2a or alpha 2b plus ribavirin over palliative care for all willingness to pay values. In addition, the combination of pegylated interferon alpha 2a and ribavirin was presented a higher probability to cost-effectiveness than that of pegylated interferon alpha 2b and ribavirin.

## Discussion

The findings have shown that the treatment with pegylated interferon alpha 2a or alpha 2b plus ribavirin had lower costs and higher outcomes since early HCV infection treatment will decrease complications and death from decompensated cirrhosis, compensated cirrhosis and HCC. These results were relevant with the study of Gerkens [16] that showed cost-effectiveness of pegylated interferon alpha 2a plus ribavirin in the treatment of CHC genotypes 1 and 6 comparing to the palliative care in Belgium and the study of Gheorghe [17] that showed cost-effectiveness of pegylated interferon alpha 2a plus ribavirin over pegylated interferon alpha 2b, and standard interferon and ribavirin combination in Romania.

For the treatment of CHC genotype 6 with pegylated interferon alpha 2a or alpha 2b plus ribavirin, the results revealed the superior of outcome to HCV infection and inferior of total treatment costs than CHC genotype 1. Despite the fact, the treatment in CHC genotype 6 had more cost-effectiveness than that of CHC genotype 1, the ICER results of both genotypes indicated pegylated interferon alpha 2a or alpha 2b plus ribavirin were dominant comparing to palliative care.

There were limitations for this study need to be reported. First, due to a lack of randomized controlled trial to compare pegylated interferon alpha 2a plus ribavirin and alpha 2b plus ribavirin in CHC genotype 6 patients, the SVR and RVR rate were calculated using the relative risk (RR) in HCV genotype 1 versus HCV genotype 6 patients from a prospective study in Thailand. Although, these rates may not indicate the exact values for genotype 6 patients, they were accepted from the expert consultation meeting and the Subcommittee for Development of the NLEM. Second, the patients who were not achieved SVR and RVR were assumed as treatment failure. We applied the number of patients who were discontinued the treatment because of virological response for null response patients and the rest of treatment failure were partial response. This may not be the exact number of those groups of patients but the number was assumed to estimate cost of treatment. Thirdly, this study assumed that the patients are 100 % complied with the treatment throughout 24 or 48 weeks. In the actual circumstances, the patients may not abide by 100 % with treatment [36], which may affect the lower effectiveness. Last, the utility score of patients in each health state was obtained from studies in other countries that may be different from Thai patients. It is noted that Thai patients’ utility score is needed for further study.

Given that this modeling study was performed before the current era of direct-acting antiviral (DAA) therapies, and DAAs are not currently available in Thailand, our study examined the available standard of care for HCV treatment in Thailand. Recently, sofosbuvir, a new antiviral drug has been approved in the US and Europe for treating CHC patients. AASLD and EASL guidelines have changed the standard treatment of HCV infection into DAAs. Although DAA showed the superior effects than pegylated interferon [37], this study did not include any DAAs in the model since the study was conducted before the drug approval. However, the study is certainly valuable to support an evidence for other developing countries who are considering the combination of pegylated interferon and ribavirin into their national drug lists. In addition, this Markov model is the first approval model for HCV treatment in Thailand. The model was validated for CHC patients along with the THASL guidelines [12], which are comparable to other guidelines [8, 10, 11]. Therefore, the model can be applied to other countries and other novel medications of HCV infection treatment. Not only is the application of the model utilized, but also the acceptance of Health Technology Assessment (HTA) for national policy making is a crucial model for other countries.

## Conclusions

The treatment of CHC infection genotypes 1 and 6 with pegylated interferon alpha 2a or alpha 2b plus ribavirin comparing to the palliative care showed cost-saving in Thailand. As cost-saving results, our study proposed the Subcommittee for Development of the NLEM to include both pegylated interferon alpha 2a and alpha 2b into the NLEM. Successively, the Subcommittee for Development of the NLEM approved to include both pegylated interferon alpha 2a and alpha 2b into the NLEM in 2014.

## Abbreviations

CHC, chronic hepatitis C virus infection; CPI, consumer price index; EVR, early virological response; HCC, hepatocellular carcinoma; HCV, hepatitis C virus infection; ICER, incremental cost-effectiveness ratio; NLEM, national list of essential medicines; NR, null response; PR, partial nonresponse; PSA, probabilistic sensitivity analysis; QALY, quality-adjusted life year; RVR, rapid virological response; SVR, sustained virological response; THB, Thai baht

## References

[1] World Health Organization. Hepatitis. 2014. http://apps.who.int/gb/ebwha/pdf_files/WHA67/A67_R6-en.pdf. Accessed 15 Oct 2014.

[2] Centers for Disease Control and Prevention. Hepatitis C: General information. 2010. http://www.cdc.gov/hepatitis/hcv/pdfs/hepcgeneralfactsheet.pdf. Accessed 20 Apr 2014.

[3] World Health Organization. Hepatitis C. 2013. http://www.who.int/mediacentre/factsheets/fs164/en/. Accessed 12 Apr 2013.

[4] Mohd Hanafiah K, Groeger J, Flaxman AD, Wiersma ST (2013). Global epidemiology of hepatitis C virus infection: new estimates of age-specific antibody to HCV seroprevalence. Hepatology.

[5] Sievert W, Altraif I, Razavi HA, Abdo A, Ahmed EA, AlOmair A, et al. A systematic review of hepatitis C virus epidemiology in Asia, Australia and Egypt. Liver International. 2011;31(s2):61–80. 10.1111/j.1478-3231.2011.02540.x 21651703

[6] El Khoury AC, Klimack WK, Wallace C, Razavi H (2012). Economic burden of hepatitis C-associated diseases in the United States. J Viral Hepat.

[7] El Khoury AC, Wallace C, Klimack WK, Razavi H (2012). Economic burden of hepatitis C-associated diseases: Europe, Asia Pacific, and the Americas. J Med Econ.

[8] Omata M, Kanda T, Yu ML, Yokosuka O, Lim SG, Jafri W (2012). APASL consensus statements and management algorithms for hepatitis C virus infection. Hepatol Int.

[9] Bunchorntavakul C, Chavalitdhamrong D, Tanwandee T (2013). Hepatitis C genotype 6: A concise review and response-guided therapy proposal. World J Hepatol.

[10] Ghany MG, Strader DB, Thomas DL, Seeff LB (2009). Diagnosis, management, and treatment of hepatitis C: an update. Hepatology.

[11] Eurapean Association for the Study of the Liver (2014). EASL Clinical Practice Guidelines: management of hepatitis C virus infection. J Hepatol.

[12] Thai Association for the Study of the Liver (2012). Thailand practice guideline for management of chronic hepatitis B and C 2012.

[13] Werayingyong P, Teerawattananon Y, International Health Policy Program (IHPP) and Health Intervention and Technology Assessment Program (HITAP) (2011). Cost-utility and budget impact of the chronic hepatitis C treatment. Research for development of health benefit package under universal health care coverage scheme: Issue 1.

[14] Nguyen NH, VuTien P, Garcia RT, Trinh H, Nguyen H, Nguyen K (2010). Response to pegylated interferon and ribavirin in Asian American patients with chronic hepatitis C genotypes 1 vs 2/3 vs 6. J Viral Hepat.

[15] Siebert U, Sroczynski G, Rossol S, Wasem J, Ravens-Sieberer U (2003). Cost effectiveness of peginterferon alpha-2b plus ribavirin versus interferon alpha-2b plus ribavirin for initial treatment of chronic hepatitis C. Gut.

[16] Gerkens S, Nechelput M, Annemans L, Peraux B, Mouchart M, Beguin C (2007). A health economic model to assess the cost-effectiveness of PEG IFN alpha-2a and ribavirin in patients with mild chronic hepatitis C. J Viral Hepat.

[17] Gheorghe L, Baculea S (2010). Cost-effectiveness of peginterferon alpha-2a and peginterferon alpha-2b combination regimens in genotype-1 naive patients with chronic hepatitis C. Hepatogastroenterology.

[18] Sullivan SD, Jensen DM, Bernstein DE, Hassanein TI, Foster GR, Lee SS (2004). Cost-effectiveness of combination peginterferon alpha-2a and ribavirin compared with interferon alpha-2b and ribavirin in patients with chronic hepatitis C. Am J Gastroenterol.

[19] Fonseca MC, Araujo GT, Araujo DV (2009). Cost effectiveness of peginterferon alfa-2B combined with ribavirin for the treatment of chronic hepatitis C in Brazil. Braz J Infect Dis.

[20] Barros FMR, Cheinquer H, Tsuchiya CT, Santos EAV (2013). Cost-effectiveness analysis of treatment with peginterferon-alfa-2a versus peginterferon-alfa-2b for patients with chronic hepatitis C under the public payer perspective in Brazil. Cost Eff Resour Alloc.

[21] Permsuwan U, Guntawongwan K, Buddhawongsa P (2008). Handling time in economic evaluation studies. J Med Assoc Thai.

[22] Flori N, Funakoshi N, Duny Y, Valats JC, Bismuth M, Christophorou D (2013). Pegylated interferon-alpha2a and ribavirin versus pegylated interferon-alpha2b and ribavirin in chronic hepatitis C: a meta-analysis. Drugs.

[23] Bureau of Policy and Strategy, Ministry of Public Health. Public health statistics 2011. Bangkok: The War Veterans Organization of Thailand Printing House. 2012. p. 58–61.

[24] Krahn M, Wong JB, Heathcote J, Scully L, Seeff L (2004). Estimating the prognosis of hepatitis C patients infected by transfusion in Canada between 1986 and 1990. Med Decis Making.

[25] Omland LH, Krarup H, Jepsen P, Georgsen J, Harritshoj LH, Riison K (2010). Mortality in patients with chronic and cleared hepatitis C viral infection: a nationwide cohort study. J Hepatol.

[26] Fattovich G, Giustina G, Degos F, Tremolada F, Diodati G, Almasio P (1997). Morbidity and mortality in compensated cirrhosis type C: a retrospective follow-up study of 384 patients. Gastroenterology.

[27] Planas R, Balleste B, Alvarez MA, Rivera M, Montoliu S, Galeras JA (2004). Natural history of decompensated hepatitis C virus-related cirrhosis. A study of 200 patients. J Hepatol.

[28] Sithinamsuwan P, Piratvisuth T, Tanomkiat W, Apakupakul N, Tongyoo S (2000). Review of 336 patients with hepatocellular carcinoma at Songklanagarind Hospital. World J Gastroenterol.

[29] McHutchison JG, Lawitz EJ, Shiffman ML, Muir AJ, Galler GW, McCone J (2009). Peginterferon alfa-2b or alfa-2a with ribavirin for treatment of hepatitis C infection. N Engl J Med.

[30] Rumi MG, Aghemo A, Prati GM, D'Ambrosio R, Donato MF, Soffredini R (2010). Randomized study of peginterferon-alpha2a plus ribavirin vs peginterferon-alpha2b plus ribavirin in chronic hepatitis C. Gastroenterology.

[31] Miyase S, Haraoka K, Ouchida Y, Morishita Y, Fujiyama S (2012). Randomized trial of peginterferon alpha-2a plus ribavirin versus peginterferon alpha-2b plus ribavirin for chronic hepatitis C in Japanese patients. J Gastroenterol.

[32] Tangkijvanich P, Komolmit P, Mahachai V, Poovorawan K, Akkarathamrongsin S, Poovorawan Y (2012). Response-guided therapy for patients with hepatitis C virus genotype 6 infection: a pilot study. J Viral Hepat.

[33] Thongsawat S, Piratvisuth T, Pramoolsinsap C, Chutaputti A, Tanwandee T, Thongsuk D (2014). Resource Utilization and Direct Medical Costs of Chronic Hepatitis C in Thailand: A Heavy but Manageable Economic Burden. Value Health Reg Issues.

[34] Riewpaiboon A. Standard Cost List for Health Technology Assessment. 2010. http://costingmenu.hitap.net/. Accessed 10 Nov 2013.

[35] Ministry of Public Health. Drug and medical supply information center. 2013. http://dmsic.moph.go.th/dmsic/index.php?p=1&id=1. Accessed 10 Nov 2013.

[36] Piratvisuth T, Tanwandee T, Mahachai V, Chutaputti A, Thongsawat S, Khow-Ean U (2004). The efficacy of peginterferon alfa-2b plus ribavirin combination in Thai patients with chronic hepatitis c. Thai J Gastroenterol.

[37] Yang HJ, Ryoo JY, Yoo BK (2015). Meta-analysis of the efficacy and safety of sofosbuvir for the treatment of hepatitis C virus infection. Int J Clin Pharm.

